# Supplementary material for: Frequency and preventative interventions for non-suicidal self-injury and suicidal behaviour in primary school-age children: a scoping review protocol
Source: BMJ Open. 2017 Jul 10;7(7):e017291. doi: 10.1136/bmjopen-2017-017291 (PMC5726135; doi:10.1136/bmjopen-2017-017291)
Supplement: Author's manuscript [file bmjopen-2017-017291.draft_revisions.pdf]

BMJ Open

Frequency and preventative interventions for non-suicidal self-injury and suicidal behaviour in primary school-aged children: a scoping review protocol

Journal:	*BMJ Open*
Manuscript ID	bmjopen-2017-017291
Article Type:	Protocol
Date Submitted by the Author:	12-Apr-2017
Complete List of Authors:	Bem, Danai; University of Birmingham, Institute of Applied Health Research<br>Connor, Charlotte ; Warwick Medical School, University of Warwick, Gibbet Hill Campus, Coventry, CV4 7AL<br>Palmer, Colin; Warwick Medical School, University of Warwick, Gibbet Hill Campus, Coventry, CV4 7AL, Mental Health and Wellbeing<br>Channa, Sunita; Warwick Medical School, University of Warwick, Gibbet Hill Campus, Coventry, CV4 7AL, Mental Health and Wellbeing<br>Birchwood, Max; University of Warwick, Division of mental health & well-being
**Primary Subject Heading**:	Mental health
Secondary Subject Heading:	Public health
Keywords:	Non-suicidal self-injury, suicide, primary schools, scoping review, intervention, prevalence

SCHOLARONE™ Manuscripts

For peer review only

TITLE

Frequency and preventative interventions for non-suicidal self-injury and suicidal behaviour in primary school-aged children: a scoping review protocol

AUTHORS

Danai Bem[1], Charlotte Connor[2*], Colin Palmer[2], Sunita Channa[2] and Max Birchwood[2]

[1]Institute of Applied Health Research, College of Medical & Dental Sciences, University of Birmingham, Edgbaston, Birmingham B15 2TT, UK

[2]Mental Health and Wellbeing, Warwick Medical School, University of Warwick, Gibbet Hill Campus, Coventry, CV4 7AL, UK

*Corresponding author:

Charlotte Connor

Mental Health and Wellbeing

Warwick Medical School

University of Warwick

Gibbet Hill Campus

Coventry

CV4 7AL

UK

Email: charlotte.connor@warwick.ac.uk

KEYWORDS

Non-suicidal self-injury, suicide, primary schools, prevalence, intervention, scoping review

WORD COUNT

2454 words (excluding title page, abstract, references, figures and tables).

For peer review only - http://bmjopen.bmj.com/site/about/guidelines.xhtml

ABSTRACT

Introduction: Non-suicidal self-injury (NSSI) and suicidal behaviour has been witnessed in children as young as 6-7 years of age, but whilst there are many reviews of preventative interventions for NSSI and suicide in adolescents, few have explored its prevalence in younger children and the potential impact of preventative interventions at this stage of life. NSSI and suicidal behaviour is an increasing concern in schools but school-based programmes can improve knowledge, attitudes and help-seeking behaviours and help prevent escalation of NSSI and later suicide. This scoping review will aim to explore the nature and extent of the evidence on the magnitude of NSSI and suicidal behaviour in primary school children, and to examine whether there are any primary school-based interventions available for the prevention of this phenomenon in 5-11 year olds.

Methods and analysis: A scoping review will be conducted using established methodology by Arksey and O'Malley and the Joanna Briggs Institute. Multiple bibliographic and indexing databases and grey literature will be searched using a combination of text words and index terms relating to NSSI, suicide, primary schools, frequency and intervention. Two reviewers will independently screen eligible studies for study selection and extract relevant data from included studies. A narrative summary of evidence will be conducted for all included studies with results presented in tables and/or diagrams. Inductive content analysis will be used to understand any narrative findings within the included studies.

Ethics and dissemination: Ethical approval is not required for this scoping review. The results of this review will be disseminated though publication in a peer-reviewed journal and presented at relevant conferences.

STRENGTHS AND LIMITATIONS OF THIS REVIEW

- To the best of our knowledge there is no comprehensive review looking at the magnitude of non-suicidal self-injury and suicidal behaviour in 5-11 year old children or any school-based interventions used for the prevention of these phenomena in this young age group.
- The proposed review will be based on established scoping review methodology, a comprehensive search, systematic screening carried out in duplicate and data abstraction employing both quantitative and qualitative approaches.
- As this is an unexplored topic relevant opinion articles, national statistics data and evidence from relevant charity and patient organisations will be considered for inclusion in an attempt to capture all available evidence in the literature.

For peer review only - http://bmjopen.bmj.com/site/about/guidelines.xhtml

INTRODUCTION

The National Self-Harm Network describes self-harm behaviour, or non-suicidal self-injury (NSSI), as "the act of deliberately causing harm to oneself by causing a physical injury, by putting oneself in dangerous situations and/or self-neglect". [1] Self-harming behaviours can include cutting or scratching the skin, burning/branding with cigarettes/lighters, scalding, overdose of tablets or other toxins, tying ligatures around the neck, punching oneself or other surfaces, banging limbs/head and hair pulling. [2] Common referred to as NSSI, it can also describe behaviours that may be culturally acceptable yet which lead to self-inflicted physical or psychological damage such as smoking, recreational drug use and excessive alcohol or body piercing, tattooing and body enhancement. [3] However, the latest edition of the Diagnostic and Statistical Manual of Mental Health Disorders (DSM-5) does not include these culturally acceptable behaviours in their criteria. [4] NSSI and suicidal behaviour are often grouped together and although these "acts" differ, specifically in terms of intent, NSSI has been shown to be a strong predictor of future suicide attempts in depressed young people. [5]

NSSI and suicidal behaviour is most commonly observed during adolescence [6 7] with an estimated lifetime prevalence in high school students between 20% and 46%. [8] The average age of onset is around 12 years of age [9] and seems to be more common among females than males. [10 11] NSSI may indicate a temporary period of emotional pain or distress, or deeper mental health issues which may result in suicide. Both behaviours have been suggested to function as alternative coping mechanisms for adolescents who have poor emotional regulation strategies. [12-15] Indeed, NSSI has been witnessed in children as young as 7 years of age with 7.6% of third-graders reporting self-harm engagement, [10] whereas 8.6% suicidal ideation was found among 6-9 year olds with elevated aggressive-disruptive behavior. [16] Being able to cope and manage emotions is something that increases with age, and NSSI and suicidal behaviour in young children may indeed represent a need to cope with difficult feelings with alternative methods in the absence of emotional regulation strategies.

With the majority of children spending most of their time in education, primary schools are well placed to identify younger children who may be at risk of developing NSSI and suicidal behaviour, and to implement interventions to support them in the development of strategies and skills to deal with life challenges and help prevent escalation into fully formed self-harm behaviour and potential crisis. [17] However, childhood self-harm and suicide remains a challenging topic for teachers who may not feel confident in dealing with such issues. Tools to support secondary school professionals and parents/carers are available and mainly focus on awareness and education programmes, screening, gatekeeper training, skills training, and peer leadership. [18 19] Few studies,

For peer review only - http://bmjopen.bmj.com/site/about/guidelines.xhtml

however, have explored the prevalence of NSSI and suicidal behaviour in primary school-aged children [10 16] and the impact of preventative interventions at this stage of life.

This protocol outlines a scoping review that, unlike a systematic review, won't attempt to estimate the prevalence of NSSI and suicidal behaviour; instead it will systematically examine the evidence on the magnitude of those behaviours in younger aged children and how visible these behaviours are in the primary school setting based on educational professionals' and school personnel's experiences. In addition, this review will aim to map and categorise the range of school-based interventions or programs available to prevent NSSI and suicidal behaviour either by targeting the students themselves or by educating school professionals and parents to increase their knowledge and awareness on such phenomena.

Research questions

This scoping review aims to identify the nature and extent of the evidence to address the following questions:

1. What evidence exists on the frequency of NSSI and suicidal behaviour in primary school-aged children?
2. Are these behaviours witnessed by educational professionals' and primary school personnel and to what extent?
3. What preventative interventions have been used in a primary school setting for NSSI and suicidal behaviour in younger aged children?

This protocol has been developed using the scoping review methodology proposed by Arksey and O'Malley [20] and further refined by the Joanna Briggs Institute. [21]

METHODS AND ANALYSIS

Search strategy

The following sources will be searched for primary studies:

- Bibliographic databases – MEDLINE, MEDLINE In Process, EMBASE, PsycINFO, CINAHL, The Cochrane Library (CDSR, DARE), Applied Social Sciences Index and Abstracts (ASSIA), Education Resources Information Centre (ERIC), British Education Index, Social Services Abstracts, and Social Policy and Practice
- Sciences and Social Sciences Citation Index (Web of Science) for citation searching
- The WHO International Clinical Trials Registry Platform, EPPI-Centre (TPoPHI) and ClinicalTrials.gov for ongoing studies

For peer review only - http://bmjopen.bmj.com/site/about/guidelines.xhtml

- Grey literature sources – Conference Proceedings Citation Index (Web of Science), Sociological Abstracts and relevant third sector organisation websites
- Checking of citation lists of included studies

The development of the search strategy will be a three-step process as described in the JBI guidance. [21] An initial limited search of MEDLINE and PsycINFO will be performed using keywords relating to the condition (NSSI & suicide) and the setting (primary school). Analysis of the identified terms in the title and abstracts of retrieved papers will be conducted by the primary researcher. A second stage search will be performed across all included databases using a combination of indexing and free text terms found in phase 1 with search terms and strategies optimised for each database. For the first and second research questions relating to frequency and experiences of NSSI and suicidal behaviour, the search strategy will also combine terms relating to frequency/prevalence and experiences/believes; whereas for the third research question terms relating to the intervention (e.g. universal, selective) will be used. Relevant keyword search terms are provided in Table 1. Finally, reference list and third sector organisation website searching will be undertaken to identify any additional eligible studies. There will be no restriction on language or date of publication.

Table 1. Keyword search terms

**Key concept**	**Keyword**
Condition	non-suicidal self-injury, (deliberate) self-harm, self-mutilation, self-neglect, self-cut, suicide, parasuicide, suicidal ideation, suicide attempt, suicidal behaviour
School	primary, elementary, basic, grade, preparatory, first, middle, junior
Child	child, kid, pupil, youth, youngster, junior, schoolboy, schoolgirl, schoolchild, juvenile
Frequency	frequency, prevalence, incidence, occurrence, trend, experience, believes
Intervention	universal, selective, targeted, indicated, school-based, classroom-based, curriculum-based, psychosocial, emotional, educational, gatekeeper training, skills training

EndNote X7.4 (Thomson Reuters, New York) will be used to manage the records identified from the literature search and to record decisions during the study selection process. Two reviewers will independently screen studies for eligibility using predetermined inclusion/exclusion criteria. Any

For peer review only - http://bmjopen.bmj.com/site/about/guidelines.xhtml

discrepancies between reviewers will be resolved with discussion or the input of a third reviewer where needed. Translation of non-English language articles will be undertaken if necessary.

Selection criteria

Types of studies

Any primary studies and reviews (systematic or narrative) on: 1) the frequency and school professionals' experiences of NSSI and suicidal behaviour in primary school-aged children (quantitative or qualitative evidence) and 2) on primary school-based interventions for the prevention of NSSI and suicidal behaviour will be included (quantitative evidence). In an attempt to identify all sources of evidence for such an unexplored topic, opinion articles and relevant organisation surveys, national statistics data and reports will be considered for inclusion. Article authors will be contacted if there is a need for further information or clarifications.

Population

The review will consider all studies on the frequency of the phenomenon and the available interventions for its prevention that include children between 5 to 11 years of age within a primary school setting. This age range was selected taking into account the variety of primary education systems found in different countries. Studies on interventions that target teachers, principals or other school-related staff (e.g. nurses, local community voluntary workers, governors) as well as parents, families and carers of 5- to 11-year olds will also be included as long as they are delivered in the primary school setting.

The included studies could potentially focus on a range of vulnerable groups including: looked-after children; young carers; children that have experienced traumatic events (e.g. emotional, physical or sexual abuse, bullying); learners with Special Educational Needs/Learning Difficulties and Disabilities; children with behaviour and attendance issues; children with mental health issues and chronic illnesses; children of parents with mental health issues; children of asylum seekers, refugees and new migrants; and Gypsy, Roma and Traveler pupils. Studies with mixed populations (e.g. young children and adolescents) will be considered if they include results for the population of interest.

Concept

For the first research question all quantitative study designs reporting the frequency of NSSI and suicidal behaviour in 5- to 11-year old children will be included. Measures of frequency will include (but not limited to) prevalence, incidence, trends and population at risk estimates.

For peer review only - http://bmjopen.bmj.com/site/about/guidelines.xhtml

Any qualitative study design that investigates the experiences of educational professionals' (e.g. educational psychologists, speech and language therapists, pediatricians) and primary school personnel (e.g. teachers, nurses, local community voluntary workers) for NSSI and suicidal behaviour in primary school settings will be included.

For the third research question any primary school-based interventions for preventing NSSI and suicidal behaviour will be included. Educational settings with mixed populations of young children and adolescents (e.g. middle schools) will be considered providing results for the population of interest can be extracted. Interventions included will be: (1) universal interventions targeted at all pupils and/or school staff, families, parents and carers (e.g. promoting health and well-being, managing feelings, gatekeeper training, mental health awareness and skills training programs); (2) selective interventions to prevent/reduce development of problems in pupils identified at risk; and (3) indicated interventions in order to prevent/reduce transition to serious health problems in pupils already presenting with low level NSSI or show suicidal behaviours. Interventions may be delivered by school staff, other health professionals or third sector organisations in the primary school setting.

Context

Any primary or elementary education setting (e.g. primary school, elementary school, lower primary school, first school, grade school) will be included. Three-tier educational settings where primary education starts at lower/first school (age range 5-9; depending on each country's system) and continues through the first grades of middle school (age range 9-13) will be considered on the bases that the reported data of interest is identifiable and extractable. Alternative educational settings (e.g. Pupil Referral Units), special schools and home-schooling will be excluded.

Charting the data

Charting will be conducted by one reviewer using a draft data charting form developed in line with the questions of the scoping review. Recorded information will be checked for accuracy by a second reviewer and any discrepancies will be resolved through discussion or referral to a third reviewer. The following data fields will be recorded from included studies: (1) general information including author, year of publication, country of origin, study design, setting, NSSI and suicidal behaviour definition; (2) population demographics for children sample (e.g. age, gender, relation with any vulnerable groups); (3) population demographics for individuals describing experiences with NSSI and suicidal behaviour; (4) type of intervention, number of sessions, who delivered it, who is the targeted population; (5) data collection method, findings (numerical for quantitative studies or authors' themes and interpretations for qualitative studies), methods of data analysis. This list is

For peer review only - http://bmjopen.bmj.com/site/about/guidelines.xhtml

indicative only as the charting process is iterative and the data extraction form may be further refined and updated at the review stage. [21] While the type of study design and methodology used will be listed, we are not excluding articles by study design, nor will we assess quality of evidence as the main aim of the scoping review is to have a broad knowledge of the available evidence and not present a view regarding the 'weight' of evidence (as recommended by Arksey and O'Malley [20]).

Collating, summarising and reporting the results

A narrative summary of evidence will be conducted for all included studies. The study selection process will be illustrated using a flow diagram similar to the Preferred Reporting Items for Systematic Reviews and Meta-Analysis flow diagram including a numerical overview of the type of the included studies. Results from quantitative studies relating to the first (frequency) and third (interventions) review questions will be presented in tables and/or diagrams in order to map: the geographical distribution of studies; the range of study population (e.g. age, socio-economic status, vulnerable groups); measures of frequency used; the range of interventions delivered in primary schools and the measures of effectiveness used; the research methods adopted.

For the second review question (experiences) tables of descriptive characteristics of the included qualitative studies will be generated mapping: the geographical distribution of studies; the range of respondents; themes and subthemes reported by authors; the research methods adopted for data analysis. Inductive content analysis will be undertaken to analyse any narrative data in included qualitative studies and opinion articles. Common categories and themes within these categories will be identified and compared with those reported by the study authors. This approach to qualitative data analysis will allow the reviewer to gain information from the studies without applying predetermined codes or theoretical perspectives. [22] The results may also be grouped according to the type of the respondent (e.g. educational professional, teaching staff).

Thus, the objective of this scoping review will not be to synthesise research findings in depth by employing meta-analysis or metasynthesis techniques but to visualise the range of evidence available in order to identify existing gaps in the literature, and reveal potential topics for future systematic reviews in the area of NSSI and suicidal behaviour during early childhood. It is hoped that this review will increase our knowledge of and help inform strategies to address this problem.

CONCLUSION

This scoping review aims to examine the evidence that exists on the frequency of NSSI and suicidal behaviour in primary school children, and to explore whether there are any primary school-based interventions available for the prevention of NSSI and suicidal behaviour in younger aged children.

For peer review only - http://bmjopen.bmj.com/site/about/guidelines.xhtml

Early childhood NSSI and suicidal behaviour has emerged as a critical issue in the last couple of decades but, as a phenomenon, is still relatively unexplored. This scoping review has the potential to identify gaps between level of need and relevant preventative measures adopted by schools in order to raise awareness, and may also contribute to the development of novel interventions for the prevention of NSSI and suicidal behaviour in this young age group.

Ethics and dissemination

This scoping review does not require ethical approval as primary data will not be collected. This protocol could not be prospectively registered with PROSPERO as scoping reviews are currently excluded. The results of this review will be disseminated though publication in a peer-reviewed journal and presented at relevant national and international conferences.

Authors' contributions

CC and MB conceived the idea for the study. All authors collaboratively designed the study. DB led the development of the search strategy. CC and DB led the writing of the protocol. CP and SC critically reviewed the protocol. All authors approved the final version of this article.

Funding statement

This study is funded by the National Institute for Health Research (NIHR) Collaboration for Leadership in Applied Health Research and Care (CLAHRC) West Midlands. The views expressed are those of the authors and not necessarily those of the NHS, the NIHR or the Department of Health.

Competing interests

None declared.

REFERENCES

1. The National Self Harm Network. What is Self-Harm? 2008. htttp://www.nshn.co.uk/What_is_Self_Harm (accessed Dec 2016).
2. Brophy M, Holmstrom R. Truth Hurts: Report of the national inquiry into self-harm among young people. Fact or Fiction? Mental Health Foundation, 2006. https://www.mentalhealth.org.uk/sites/default/files/truth_hurts (accessed Dec 2016).
3. Favazza AR. The coming of age of self-mutilation. *J Nerv Ment Dis* 1998;**186**(5):259-68.
4. American Psychiatric Association. *Diagnostic and Statistical Manual of Mental Disorders (DSM–5)*. Washington DC: American Psychiatric Association, 2013.

For peer review only - http://bmjopen.bmj.com/site/about/guidelines.xhtml

5. Wilkinson P, Goodyer I. Non-suicidal self-injury. *Eur Child Adolesc Psychiatry* 2011;**20**(2):103-8.

6. Hawton K, Saunders KE, O'Connor RC. Self-harm and suicide in adolescents. *Lancet* 2012;**379**(9834):2373-82.

7. Plener PL, Schumacher TS, Munz LM, et al. The longitudinal course of non-suicidal self-injury and deliberate self-harm: a systematic review of the literature. *Borderline Personal Disord Emot Dysregul* 2015;**2**:2.

8. Nixon MK, Heath NL. *Self-injury in youth: The essential guide to assessment and intervention*. New York: Routledge, 2008.

9. Fox C, Hawton K. *Deliberate self-harm in adolescence*. London: Jessica Kingsley, 2004.

10. Barrocas AL, Hankin BL, Young JF, et al. Rates of nonsuicidal self-injury in youth: age, sex, and behavioral methods in a community sample. *Pediatrics* 2012;**130**(1):39-45.

11. Moran P, Coffey C, Romaniuk H, et al. The natural history of self-harm from adolescence to young adulthood: a population-based cohort study. *Lancet* 2012;**379**(9812):236-43.

12. Gratz KL, Roemer L. The relationship between emotion dysregulation and deliberate self-harm among female undergraduate students at an urban commuter university. *Cogn Behav Ther* 2008;**37**(1):14-25.

13. Klonsky ED. The functions of self-injury in young adults who cut themselves: clarifying the evidence for affect-regulation. *Psychiatry Res* 2009;**166**(2-3):260-8.

14. Palmer B, Martin, G. Self-Harm in children under 14: A comparison of inpatients who self-harm with those who do not. *J Child Adolesc Behav* 2016;**4**:302.

15. Zimmerman P, Iwanski A. Emotion regulation from early adolescence to emerging adulthood and middle adulthood: Age differences, gender differences, and emotion specific developmental variations. *Int J Behav Dev* 2014;**38**(2):182-94.

16. Wyman PA, Gaudieri PA, Schmeelk-Cone K, et al. Emotional triggers and psychopathology associated with suicidal ideation in urban children with elevated aggressive-disruptive behavior. *J Abnorm Child Psychol* 2009;**37**(7):917-28.

17. Alfonso ML, Kaur R. Self-injury among early adolescents: identifying segments protected and at risk. *J Sch Health* 2012;**82**(12):537-47.

18. Joshi SV, Hartley SN, Kessler M, et al. School-based suicide prevention: content, process, and the role of trusted adults and peers. *Child Adolesc Psychiatr Clin N Am* 2015;**24**(2):353-70.

19. Robinson J, Cox G, Malone A, et al. A systematic review of school-based interventions aimed at preventing, treating, and responding to suicide- related behavior in young people. *Crisis* 2013;**34**(3):164-82.

For peer review only

For peer review only - http://bmjopen.bmj.com/site/about/guidelines.xhtml

20. Arksey H, O'Malley L. Scoping studies: towards a methodological framework. *Int J Soc Res Methodol* 2005;**8**(1):19-32.

21. Joanna Briggs Institute. *Joanna Briggs Institute Reviewers' Manual 2015: Methodology for JBI Scoping Reviews*. Australia: The Joanna Briggs Institute, 2015.

22. Hsieh HF, Shannon SE. Three approaches to qualitative content analysis. *Qual Health Res* 2005;**15**(9):1277-88.

For peer review only

For peer review only - http://bmjopen.bmj.com/site/about/guidelines.xhtml

PRISMA-P checklist

Section/topic	Item #	Checklist item	Reported on page
**ADMINISTRATIVE INFORMATION**			
**Title**			
**Identification**	1a	Identify the report as a protocol of a systematic review	1 (Title page)
**Update**	1b	If the protocol is for an update of a previous systematic review, identify as such	N/A
**Registration**	2	If registered, provide the name of the registry (e.g., PROSPERO) and registration number	N/A for scoping reviews
**Authors**			
**Contact**	3a	Provide name, institutional affiliation, and e-mail address of all protocol authors; provide physical mailing address of corresponding author	1
**Contributions**	3b	Describe contributions of protocol authors and identify the guarantor of the review	9
**Amendments**	4	If the protocol represents an amendment of a previously completed or published protocol, identify as such and list changes; otherwise, state plan for documenting important protocol amendments	N/A
**Support**			
**Sources**	5a	Indicate sources of financial or other support for the review	9
**Sponsor**	5b	Provide name for the review funder and/or sponsor	9
**Role of sponsor/funder**	5c	Describe roles of funder(s), sponsor(s), and/or institution(s), if any, in developing the protocol	9
**INTRODUCTION**			
**Rationale**	6	Describe the rationale for the review in the context of what is already known	3-4
**Objectives**	7	Provide an explicit statement of the question(s) the review will address with reference to participants, interventions, comparators, and outcomes (PICO)	4
**METHODS**			
**Eligibility criteria**	8	Specify the study characteristics (e.g., PICO, study design, setting, time frame) and report characteristics (e.g., years considered, language, publication status) to be used as criteria for eligibility for the review	6-7
**Information sources**	9	Describe all intended information sources (e.g., electronic databases, contact with study authors, trial registers, or other grey literature sources) with planned dates of coverage	4-5

**Search strategy**	10	Present draft of search strategy to be used for at least one electronic database, including planned limits, such that it could be repeated	5
**Study records**			
**Data management**	11a	Describe the mechanism(s) that will be used to manage records and data throughout the review	5-6
**Selection process**	11b	State the process that will be used for selecting studies (e.g., two independent reviewers) through each phase of the review (i.e., screening, eligibility, and inclusion in meta-analysis)	5-7
**Data collection process**	11c	Describe planned method of extracting data from reports (e.g., piloting forms, done independently, in duplicate), any processes for obtaining and confirming data from investigators	7-8
**Data items**	12	List and define all variables for which data will be sought (e.g., PICO items, funding sources), any pre-planned data assumptions and simplifications	7-8
**Outcomes and prioritization**	13	List and define all outcomes for which data will be sought, including prioritization of main and additional outcomes, with rationale	6-8
**Risk of bias in individual studies**	14	Describe anticipated methods for assessing risk of bias of individual studies, including whether this will be done at the outcome or study level, or both; state how this information will be used in data synthesis	N/A for scoping reviews
**Data**			
**Synthesis**	15a	Describe criteria under which study data will be quantitatively synthesized	8
	15b	If data are appropriate for quantitative synthesis, describe planned summary measures, methods of handling data, and methods of combining data from studies, including any planned exploration of consistency (e.g., I^2, Kendall's tau)	N/A for scoping reviews
	15c	Describe any proposed additional analyses (e.g., sensitivity or subgroup analyses, meta-regression)	N/A for scoping reviews
	15d	If quantitative synthesis is not appropriate, describe the type of summary planned	8
**Meta-bias(es)**	16	Specify any planned assessment of meta-bias(es) (e.g., publication bias across studies, selective reporting within studies)	N/A for scoping reviews
**Confidence in cumulative evidence**	17	Describe how the strength of the body of evidence will be assessed (e.g., GRADE)	N/A for scoping reviews

BMJ Open

Frequency and preventative interventions for non-suicidal self-injury and suicidal behaviour in primary school-aged children: a scoping review protocol

Journal:	*BMJ Open*
Manuscript ID	bmjopen-2017-017291.R1
Article Type:	Protocol
Date Submitted by the Author:	01-Jun-2017

Complete List of Authors:	Bem, Danai; University of Birmingham, Institute of Applied Health Research<br>Connor, Charlotte ; Warwick Medical School, University of Warwick, Gibbet Hill Campus, Coventry, CV4 7AL<br>Palmer, Colin; Warwick Medical School, University of Warwick, Gibbet Hill Campus, Coventry, CV4 7AL, Mental Health and Wellbeing<br>Channa, Sunita; Warwick Medical School, University of Warwick, Gibbet Hill Campus, Coventry, CV4 7AL, Mental Health and Wellbeing<br>Birchwood, Max; University of Warwick, Division of mental health & well-being

**Primary Subject Heading**:	Mental health
Secondary Subject Heading:	Public health, Epidemiology
Keywords:	Non-suicidal self-injury, suicide, primary schools, scoping review, intervention, prevalence

SCHOLARONE™
Manuscripts

TITLE

Frequency and preventative interventions for non-suicidal self-injury and suicidal behaviour in primary school-aged children: a scoping review protocol

AUTHORS

Danai Bem[1], Charlotte Connor[2*], Colin Palmer[2], Sunita Channa[2] and Max Birchwood[2]

[1]Institute of Applied Health Research, College of Medical & Dental Sciences, University of Birmingham, Edgbaston, Birmingham B15 2TT, UK

[2]Mental Health and Wellbeing, Warwick Medical School, University of Warwick, Gibbet Hill Campus, Coventry, CV4 7AL, UK

*Corresponding author:

Charlotte Connor

Mental Health and Wellbeing

Warwick Medical School

University of Warwick

Gibbet Hill Campus

Coventry

CV4 7AL

UK

Email: charlotte.connor@warwick.ac.uk

KEYWORDS

Non-suicidal self-injury, suicide, primary schools, prevalence, intervention, scoping review

WORD COUNT

2469 words (excluding title page, abstract, references, figures and tables).

For peer review only

ABSTRACT

Introduction: Non-suicidal self-injury (NSSI) and suicidal behaviour has been witnessed in children as young as 6-7 years of age, but whilst there are many reviews of preventative interventions for NSSI and suicide in adolescents, few have explored its prevalence in younger children and the potential impact of preventative interventions at this stage of life. NSSI and suicidal behaviour is an increasing concern in schools but school-based programmes can improve knowledge, attitudes and help-seeking behaviours and help prevent escalation of NSSI and later suicide. This scoping review will aim to explore the nature and extent of the evidence on the magnitude of NSSI and suicidal behaviour in primary school children, and to examine whether there are any primary school-based interventions available for the prevention of this phenomenon in 5-11 year olds.

Methods and analysis: A scoping review will be conducted using established methodology by Arksey and O'Malley and the Joanna Briggs Institute. Multiple bibliographic and indexing databases and grey literature will be searched using a combination of text words and index terms relating to NSSI, suicide, primary schools, frequency and intervention. Two reviewers will independently screen eligible studies for study selection and extract relevant data from included studies. A narrative summary of evidence will be conducted for all included studies with results presented in tables and/or diagrams. Inductive content analysis will be used to understand any narrative findings within the included studies.

Ethics and dissemination: Ethical approval is not required for this scoping review. The results of this review will be disseminated though publication in a peer-reviewed journal and presented at relevant conferences.

STRENGTHS AND LIMITATIONS OF THIS REVIEW

- To the best of our knowledge there is no comprehensive review looking at the magnitude of non-suicidal self-injury and suicidal behaviour in 5-11 year old children or any school-based interventions used for the prevention of these phenomena in this young age group.
- The proposed review will be based on established scoping review methodology, a comprehensive search, systematic screening carried out in duplicate and data abstraction employing both quantitative and qualitative approaches.
- As this is an unexplored topic relevant opinion articles, national statistics data and evidence from relevant charity and patient organisations will be considered for inclusion in an attempt to capture all available evidence in the literature.

For peer review only - http://bmjopen.bmj.com/site/about/guidelines.xhtml

INTRODUCTION

The National Self-Harm Network describes self-harm behaviour, or non-suicidal self-injury (NSSI), as "the act of deliberately causing harm to oneself by causing a physical injury, by putting oneself in dangerous situations and/or self-neglect". [1] Self-harming behaviours can include cutting or scratching the skin, burning/branding with cigarettes/lighters, scalding, overdose of tablets or other toxins, tying ligatures around the neck, punching oneself or other surfaces, banging limbs/head and hair pulling. [2] Common referred to as NSSI, it can also describe behaviours that may be culturally acceptable yet which lead to self-inflicted physical or psychological damage such as smoking, recreational drug use and excessive alcohol or body piercing, tattooing and body enhancement. [3] However, the latest edition of the Diagnostic and Statistical Manual of Mental Health Disorders (DSM-5) does not include these culturally acceptable behaviours in their criteria. [4] NSSI and suicidal behaviour are often grouped together and although these "acts" differ, specifically in terms of intent, NSSI has been shown to be a strong predictor of future suicide attempts in depressed young people. [5]

NSSI and suicidal behaviour is most commonly observed during adolescence [6 7] with an estimated lifetime prevalence in high school students between 20% and 46%. [8] The average age of onset is around 12 years of age [9] and seems to be more common among females than males. [10 11] NSSI may indicate a temporary period of emotional pain or distress, or deeper mental health issues which may result in suicide. Both behaviours have been suggested to function as alternative coping mechanisms for adolescents who have poor emotional regulation strategies. [12-15] Indeed, NSSI has been witnessed in children as young as 7 years of age with 7.6% of third-graders reporting self-harm engagement, [10] whereas 8.6% suicidal ideation was found among 6-9 year olds with elevated aggressive-disruptive behavior. [16] Being able to cope and manage emotions is something that increases with age, and NSSI and suicidal behaviour in young children may indeed represent a need to cope with difficult feelings with alternative methods in the absence of emotional regulation strategies.

With the majority of children spending most of their time in education, primary schools are well placed to identify younger children who may be at risk of developing NSSI and suicidal behaviour, and to implement interventions to support them in the development of strategies and skills to deal with life challenges and help prevent escalation into fully formed self-harm behaviour and potential crisis. [17] However, childhood self-harm and suicide remains a challenging topic for teachers who may not feel confident in dealing with such issues. Tools to support secondary school professionals and parents/carers are available and mainly focus on awareness and education programmes, screening, gatekeeper training, skills training, and peer leadership. [18 19] Few studies,

For peer review only - http://bmjopen.bmj.com/site/about/guidelines.xhtml

however, have explored the prevalence of NSSI and suicidal behaviour in primary school-aged children [10 16] and the impact of preventative interventions at this stage of life.

This protocol outlines a scoping review that, unlike a systematic review, won't attempt to estimate the prevalence of NSSI and suicidal behaviour; instead it will systematically examine the evidence on the magnitude of those behaviours in younger aged children and how visible these behaviours are in the primary school setting based on educational professionals' and school personnel's experiences. In addition, this review will aim to map and categorise the range of school-based interventions or programs available to prevent NSSI and suicidal behaviour either by targeting the students themselves or by educating school professionals and parents to increase their knowledge and awareness on such phenomena.

Research questions

This scoping review aims to identify the nature and extent of the evidence to address the following questions:

1. What evidence exists on the frequency of NSSI and suicidal behaviour in primary school-aged children?
2. Are these behaviours witnessed by educational professionals' and primary school personnel and to what extent?
3. What preventative interventions have been used in a primary school setting for NSSI and suicidal behaviour in younger aged children?

This protocol has been developed using the scoping review methodology proposed by Arksey and O'Malley [20] and further refined by the Joanna Briggs Institute. [21]

METHODS AND ANALYSIS

Search strategy

The following sources will be searched from inception up to the date the search will be conducted for identifying relevant studies:

- Bibliographic databases – MEDLINE, MEDLINE In Process, EMBASE, PsycINFO, CINAHL, The Cochrane Library (CDSR, DARE), Applied Social Sciences Index and Abstracts (ASSIA), Education Resources Information Centre (ERIC), British Education Index, Social Services Abstracts, and Social Policy and Practice
- Sciences and Social Sciences Citation Index (Web of Science) for citation searching
- The WHO International Clinical Trials Registry Platform, EPPI-Centre (TPoPHI) and ClinicalTrials.gov for ongoing studies

For peer review only - http://bmjopen.bmj.com/site/about/guidelines.xhtml

- Grey literature sources – Conference Proceedings Citation Index (Web of Science), Sociological Abstracts and relevant third sector organisation websites
- Checking of citation lists of included studies

The development of the search strategy will be a three-step process as described in the JBI guidance. [21] An initial limited search of MEDLINE and PsycINFO will be performed using keywords relating to the condition (NSSI & suicide) and the setting (primary school). Analysis of the identified terms in the title and abstracts of retrieved papers will be conducted by the primary researcher. A second stage search will be performed across all included databases using a combination of indexing and free text terms found in phase 1 with search terms and strategies optimised for each database. For the first and second research questions relating to frequency and experiences of NSSI and suicidal behaviour, the search strategy will also combine terms relating to frequency/prevalence and experiences/believes; whereas for the third research question terms relating to the intervention (e.g. universal, selective) will be used. Relevant keyword search terms are provided in Table 1. Finally, reference list and third sector organisation website searching will be undertaken to identify any additional eligible studies. There will be no restriction on language or date of publication.

Table 1. Keyword search terms

Key concept	Keyword
Condition	(non-suicidal) self-injur*, (deliberate) self-harm*, self-mutilat*, self-neglect, self-cut*, parasuicid*, suicid* ideation/attempt/behaviour/think*/thought/plan*
School	primary, elementary, basic, grade, preparatory, first, middle, junior
Child	child*, kid, pupil, youth, youngster, junior, schoolboy, schoolgirl, schoolchild, juvenile, student
Frequency	frequency, prevalence, incidence, occurrence, trend, experience, believes, number, count, population at risk
Intervention	universal, selective, targeted, indicated, school-based, classroom-based, curriculum-based, psycho*, therap*, emotional, educational, gatekeeper training, skills training, social emotional learning

* truncation symbol/wildcard

EndNote X7.4 (Thomson Reuters, New York) will be used to manage the records identified from the literature search and to record decisions during the study selection process. Two reviewers will independently screen studies for eligibility using predetermined inclusion/exclusion criteria. Any

For peer review only - http://bmjopen.bmj.com/site/about/guidelines.xhtml

discrepancies between reviewers will be resolved with discussion or the input of a third reviewer where needed. Translation of non-English language articles will be undertaken if necessary.

Selection criteria

Types of studies

Any primary studies and reviews (systematic or narrative) on: 1) the frequency and school professionals' experiences of NSSI and suicidal behaviour in primary school-aged children (quantitative or qualitative evidence) and 2) on primary school-based interventions for the prevention of NSSI and suicidal behaviour will be included (quantitative evidence). In an attempt to identify all sources of evidence for such an unexplored topic, opinion articles and relevant organisation surveys, national statistics data and reports will be considered for inclusion. Article authors will be contacted if there is a need for further information or clarifications.

Population

The review will consider all studies on the frequency of the phenomenon and the available interventions for its prevention that include children between 5 to 11 years of age within a primary school setting. This age range was selected taking into account the variety of primary education systems found in different countries. Studies on interventions that target teachers, principals or other school-related staff (e.g. nurses, local community voluntary workers, governors) as well as parents, families and carers of 5- to 11-year olds will also be included as long as they are delivered in the primary school setting.

The included studies could potentially focus on a range of vulnerable groups including: looked-after children; young carers; children that have experienced traumatic events (e.g. emotional, physical or sexual abuse, bullying); learners with Special Educational Needs/Learning Difficulties and Disabilities; children with behaviour and attendance issues; children with mental health issues and chronic illnesses; children of parents with mental health issues; children of asylum seekers, refugees and new migrants; first nation/indigenous children; and Gypsy, Roma and Traveler pupils. Studies with mixed populations (e.g. young children and adolescents) will be considered if they include results for the population of interest.

Concept

For the first research question all quantitative study designs reporting the frequency of NSSI and suicidal behaviour in 5- to 11-year old children will be included. Measures of frequency will include (but not limited to) prevalence, incidence, trends and population at risk estimates.

For peer review only - http://bmjopen.bmj.com/site/about/guidelines.xhtml

Any qualitative study design that investigates the experiences of educational professionals' (e.g. educational psychologists, speech and language therapists, pediatricians) and primary school personnel (e.g. teachers, nurses, local community voluntary workers) for NSSI and suicidal behaviour in primary school settings will be included.

For the third research question any primary school-based interventions for preventing NSSI and suicidal behaviour will be included. Educational settings with mixed populations of young children and adolescents (e.g. middle schools) will be considered providing results for the population of interest can be extracted. Interventions included will be: (1) universal interventions targeted at all pupils and/or school staff, families, parents and carers (e.g. promoting health and well-being, managing feelings, gatekeeper training, mental health awareness and skills training programs); (2) selective interventions to prevent/reduce development of problems in pupils identified at risk; and (3) indicated interventions in order to prevent/reduce transition to serious health problems in pupils already presenting with low level NSSI or show suicidal behaviours. Interventions may be delivered by school staff, other health professionals or third sector organisations in the primary school setting.

Context

Any primary or elementary education setting (e.g. primary school, elementary school, lower primary school, first school, grade school) will be included. Three-tier educational settings where primary education starts at lower/first school (age range 5-9; depending on each country's system) and continues through the first grades of middle school (age range 9-13) will be considered on the bases that the reported data of interest is identifiable and extractable. Alternative educational settings (e.g. Pupil Referral Units), special schools and home-schooling will be excluded.

Charting the data

Charting will be conducted by one reviewer using a draft data charting form developed in line with the questions of the scoping review. Recorded information will be checked for accuracy by a second reviewer and any discrepancies will be resolved through discussion or referral to a third reviewer. The following data fields will be recorded from included studies: (1) general information including author, year of publication, country of origin, study design, setting, NSSI and suicidal behaviour definition; (2) population demographics for children sample (e.g. age, gender, relation with any vulnerable groups); (3) population demographics for individuals describing experiences with NSSI and suicidal behaviour; (4) type of intervention, number of sessions, who delivered it, who is the targeted population; (5) data collection method, findings (numerical for quantitative studies or authors' themes and interpretations for qualitative studies), methods of data analysis. This list is

For peer review only - http://bmjopen.bmj.com/site/about/guidelines.xhtml

indicative only as the charting process is iterative and the data extraction form may be further refined and updated at the review stage. [21] While the type of study design and methodology used will be listed, we are not excluding articles by study design, nor will we assess quality of evidence as the main aim of the scoping review is to have a broad knowledge of the available evidence and not present a view regarding the 'weight' of evidence (as recommended by Arksey and O'Malley). [20]

Collating, summarising and reporting the results

A narrative summary of evidence will be conducted for all included studies. The study selection process will be illustrated using a flow diagram similar to the Preferred Reporting Items for Systematic Reviews and Meta-Analysis flow diagram including a numerical overview of the type of the included studies. Results from quantitative studies relating to the first (frequency) and third (interventions) review questions will be presented in tables and/or diagrams in order to map: the geographical distribution of studies; the range of study population (e.g. age, socio-economic status, vulnerable groups); measures of frequency used; the range of interventions delivered in primary schools and the measures of effectiveness used; the research methods adopted.

For the second review question (experiences) tables of descriptive characteristics of the included qualitative studies will be generated mapping: the geographical distribution of studies; the range of respondents; themes and subthemes reported by authors; the research methods adopted for data analysis. Inductive content analysis will be undertaken to analyse any narrative data in included qualitative studies and opinion articles. Common categories and themes within these categories will be identified and compared with those reported by the study authors. This approach to qualitative data analysis will allow the reviewer to gain information from the studies without applying predetermined codes or theoretical perspectives. [22] The results may also be grouped according to the type of the respondent (e.g. educational professional, teaching staff).

Thus, the objective of this scoping review will not be to synthesise research findings in depth by employing meta-analysis or metasynthesis techniques but to visualise the range of evidence available in order to identify existing gaps in the literature, and reveal potential topics for future systematic reviews in the area of NSSI and suicidal behaviour during early childhood. It is hoped that this review will increase our knowledge of and help inform strategies to address this problem.

CONCLUSION

This scoping review aims to examine the evidence that exists on the frequency of NSSI and suicidal behaviour in primary school children, and to explore whether there are any primary school-based interventions available for the prevention of NSSI and suicidal behaviour in younger aged children.

For peer review only - http://bmjopen.bmj.com/site/about/guidelines.xhtml

Early childhood NSSI and suicidal behaviour has emerged as a critical issue in the last couple of decades but, as a phenomenon, is still relatively unexplored. This scoping review has the potential to identify gaps between level of need and relevant preventative measures adopted by schools in order to raise awareness, and may also contribute to the development of novel interventions for the prevention of NSSI and suicidal behaviour in this young age group.

Ethics and dissemination

This scoping review does not require ethical approval as primary data will not be collected. This protocol could not be prospectively registered with PROSPERO as scoping reviews are currently excluded. The results of this review will be disseminated though publication in a peer-reviewed journal and presented at relevant national and international conferences.

Authors' contributions

CC and MB conceived the idea for the study. All authors collaboratively designed the study. DB led the development of the search strategy. CC and DB led the writing of the protocol. CP and SC critically reviewed the protocol. All authors approved the final version of this article.

Funding statement

This study is funded by the National Institute for Health Research (NIHR) Collaboration for Leadership in Applied Health Research and Care (CLAHRC) West Midlands. The views expressed are those of the authors and not necessarily those of the NHS, the NIHR or the Department of Health.

Competing interests

None declared.

REFERENCES

1. The National Self Harm Network. What is Self-Harm? 2008. htttp://www.nshn.co.uk/What_is_Self_Harm (accessed Dec 2016).
2. Brophy M, Holmstrom R. Truth Hurts: Report of the national inquiry into self-harm among young people. Fact or Fiction? Mental Health Foundation, 2006. https://www.mentalhealth.org.uk/sites/default/files/truth_hurts (accessed Dec 2016).
3. Favazza AR. The coming of age of self-mutilation. *J Nerv Ment Dis* 1998;**186**(5):259-68.
4. American Psychiatric Association. *Diagnostic and Statistical Manual of Mental Disorders (DSM–5)*. Washington DC: American Psychiatric Association, 2013.

For peer review only - http://bmjopen.bmj.com/site/about/guidelines.xhtml

5. Wilkinson P, Goodyer I. Non-suicidal self-injury. *Eur Child Adolesc Psychiatry* 2011;**20**(2):103-8.

6. Hawton K, Saunders KE, O'Connor RC. Self-harm and suicide in adolescents. *Lancet* 2012;**379**(9834):2373-82.

7. Plener PL, Schumacher TS, Munz LM, et al. The longitudinal course of non-suicidal self-injury and deliberate self-harm: a systematic review of the literature. *Borderline Personal Disord Emot Dysregul* 2015;**2**:2.

8. Nixon MK, Heath NL. *Self-injury in youth: The essential guide to assessment and intervention*. New York: Routledge, 2008.

9. Fox C, Hawton K. *Deliberate self-harm in adolescence*. London: Jessica Kingsley, 2004.

10. Barrocas AL, Hankin BL, Young JF, et al. Rates of nonsuicidal self-injury in youth: age, sex, and behavioral methods in a community sample. *Pediatrics* 2012;**130**(1):39-45.

11. Moran P, Coffey C, Romaniuk H, et al. The natural history of self-harm from adolescence to young adulthood: a population-based cohort study. *Lancet* 2012;**379**(9812):236-43.

12. Gratz KL, Roemer L. The relationship between emotion dysregulation and deliberate self-harm among female undergraduate students at an urban commuter university. *Cogn Behav Ther* 2008;**37**(1):14-25.

13. Klonsky ED. The functions of self-injury in young adults who cut themselves: clarifying the evidence for affect-regulation. *Psychiatry Res* 2009;**166**(2-3):260-8.

14. Palmer B, Martin, G. Self-Harm in children under 14: A comparison of inpatients who self-harm with those who do not. *J Child Adolesc Behav* 2016;**4**:302.

15. Zimmerman P, Iwanski A. Emotion regulation from early adolescence to emerging adulthood and middle adulthood: Age differences, gender differences, and emotion specific developmental variations. *Int J Behav Dev* 2014;**38**(2):182-94.

16. Wyman PA, Gaudieri PA, Schmeelk-Cone K, et al. Emotional triggers and psychopathology associated with suicidal ideation in urban children with elevated aggressive-disruptive behavior. *J Abnorm Child Psychol* 2009;**37**(7):917-28.

17. Alfonso ML, Kaur R. Self-injury among early adolescents: identifying segments protected and at risk. *J Sch Health* 2012;**82**(12):537-47.

18. Joshi SV, Hartley SN, Kessler M, et al. School-based suicide prevention: content, process, and the role of trusted adults and peers. *Child Adolesc Psychiatr Clin N Am* 2015;**24**(2):353-70.

19. Robinson J, Cox G, Malone A, et al. A systematic review of school-based interventions aimed at preventing, treating, and responding to suicide- related behavior in young people. *Crisis* 2013;**34**(3):164-82.

For peer review only

For peer review only - http://bmjopen.bmj.com/site/about/guidelines.xhtml

20. Arksey H, O'Malley L. Scoping studies: towards a methodological framework. *Int J Soc Res Methodol* 2005;**8**(1):19-32.

21. Joanna Briggs Institute. *Joanna Briggs Institute Reviewers' Manual 2015: Methodology for JBI Scoping Reviews*. Australia: The Joanna Briggs Institute, 2015.

22. Hsieh HF, Shannon SE. Three approaches to qualitative content analysis. *Qual Health Res* 2005;**15**(9):1277-88.

For peer review only

For peer review only - http://bmjopen.bmj.com/site/about/guidelines.xhtml

PRISMA-P checklist

Section/topic	Item #	Checklist item	Reported on page
**ADMINISTRATIVE INFORMATION**			
**Title**			
**Identification**	1a	Identify the report as a protocol of a systematic review	1 (Title page)
**Update**	1b	If the protocol is for an update of a previous systematic review, identify as such	N/A
**Registration**	2	If registered, provide the name of the registry (e.g., PROSPERO) and registration number	N/A for scoping reviews
**Authors**			
**Contact**	3a	Provide name, institutional affiliation, and e-mail address of all protocol authors; provide physical mailing address of corresponding author	1
**Contributions**	3b	Describe contributions of protocol authors and identify the guarantor of the review	9
**Amendments**	4	If the protocol represents an amendment of a previously completed or published protocol, identify as such and list changes; otherwise, state plan for documenting important protocol amendments	N/A
**Support**			
**Sources**	5a	Indicate sources of financial or other support for the review	9
**Sponsor**	5b	Provide name for the review funder and/or sponsor	9
**Role of sponsor/funder**	5c	Describe roles of funder(s), sponsor(s), and/or institution(s), if any, in developing the protocol	9
**INTRODUCTION**			
**Rationale**	6	Describe the rationale for the review in the context of what is already known	3-4
**Objectives**	7	Provide an explicit statement of the question(s) the review will address with reference to participants, interventions, comparators, and outcomes (PICO)	4
**METHODS**			
**Eligibility criteria**	8	Specify the study characteristics (e.g., PICO, study design, setting, time frame) and report characteristics (e.g., years considered, language, publication status) to be used as criteria for eligibility for the review	6-7
**Information sources**	9	Describe all intended information sources (e.g., electronic databases, contact with study authors, trial registers, or other grey literature sources) with planned dates of coverage	4-5

For peer review only - http://bmjopen.bmj.com/site/about/guidelines.xhtml

**Search strategy**	10	Present draft of search strategy to be used for at least one electronic database, including planned limits, such that it could be repeated	5
**Study records**			
**Data management**	11a	Describe the mechanism(s) that will be used to manage records and data throughout the review	5-6
**Selection process**	11b	State the process that will be used for selecting studies (e.g., two independent reviewers) through each phase of the review (i.e., screening, eligibility, and inclusion in meta-analysis)	5-7
**Data collection process**	11c	Describe planned method of extracting data from reports (e.g., piloting forms, done independently, in duplicate), any processes for obtaining and confirming data from investigators	7-8
**Data items**	12	List and define all variables for which data will be sought (e.g., PICO items, funding sources), any pre-planned data assumptions and simplifications	7-8
**Outcomes and prioritization**	13	List and define all outcomes for which data will be sought, including prioritization of main and additional outcomes, with rationale	6-8
**Risk of bias in individual studies**	14	Describe anticipated methods for assessing risk of bias of individual studies, including whether this will be done at the outcome or study level, or both; state how this information will be used in data synthesis	N/A for scoping reviews
**Data**			
**Synthesis**	15a	Describe criteria under which study data will be quantitatively synthesized	8
	15b	If data are appropriate for quantitative synthesis, describe planned summary measures, methods of handling data, and methods of combining data from studies, including any planned exploration of consistency (e.g., I^2, Kendall's tau)	N/A for scoping reviews
	15c	Describe any proposed additional analyses (e.g., sensitivity or subgroup analyses, meta-regression)	N/A for scoping reviews
	15d	If quantitative synthesis is not appropriate, describe the type of summary planned	8
**Meta-bias(es)**	16	Specify any planned assessment of meta-bias(es) (e.g., publication bias across studies, selective reporting within studies)	N/A for scoping reviews
**Confidence in cumulative evidence**	17	Describe how the strength of the body of evidence will be assessed (e.g., GRADE)	N/A for scoping reviews
